# Expanding Fiscal Space for Healthcare System through Efficiency: A Qualitative Study from Iran

**Published:** 2020-04

**Authors:** Firoozeh BAIRAMI, Amirhossein TAKIAN, Ali AKBARI SARI, Iraj HARIRCHI, Minoo ALIPOURI SAKHA

**Affiliations:** 1. Department of Health Management and Economics, School of Public Health, Tehran University of Medical Sciences, Tehran, Iran; 2. Department of Global Health and Public Policy, School of Public Health, Tehran University of Medical Sciences, Tehran, Iran; 3. Health Equity Research Center (HERC), Tehran University of Medical Sciences, Tehran, Iran; 4. Cancer Institute, Tehran University of Medical Sciences, Tehran, Iran

**Keywords:** Fiscal space, Efficiency, Health financing, Iran

## Abstract

**Background:**

Healthcare systems are always facing increasing public demands to provide better services. Therefore, countries always need more resources and are constantly seeking more fiscal space for health. Freeing up resources through improving efficiency can be a practical option for all settings, particularly countries with low resources. This study aimed to identify feasible options for expanding fiscal space through efficiency within Iran’s healthcare system.

**Methods:**

This was a qualitative study. We conducted 29 semi-structured in-depth interviews with stakeholders at various levels of healthcare system in 2017 and 2018. We used mixed method (deductive and inductive) qualitative content analysis. Pre-defined themes extracted from literature and meanwhile new subthemes were developed and added to the initial framework.

**Results:**

We identified three main themes that affect the efficiency of healthcare system in Iran: administration, implementation, and monitoring. Problematic administration, inappropriate implementation and lack of good monitoring in healthcare initiatives may lead to inefficiencies and wasting resources. Recognizing these leakages in every healthcare system can free up some resources.

**Conclusion:**

Irrespective of their economic development, all countries may, to some extent, face limited resources to address ever-increasing needs in their healthcare systems. While generating new resources is not always possible, enhancing efficiency to expand fiscal space might be a feasible option. Healthcare systems should identify the leakages and respond to wastages with appropriate planning. Getting the most out of current resources is possible through proper administration, good implementation and a well-established monitoring system for healthcare initiatives.

## Introduction

Achieving sustainable health development is an ultimate goal for many governments. Nations with healthy people are more likely to be productive and prosperous. Health is also viewed as a right of all people (1, 2). As a result of ever-increasing public demand to provide better healthcare, asking more resources for health systems are inevitable (2, 3). As a core function of any healthcare system, lack of sustained and sufficient financial resources is often the main challenge of healthcare systems, particularly in low and middle-income countries (4), which may lead to high out of pocket payment, catastrophic spending on health and impoverishment (5–7). Hence, countries are constantly seeking more fiscal space for health.

Fiscal space is defined (Peter Heller, P.75) as “the capacity of government to provide additional budgetary rooms for a desired purpose without any prejudice to the sustainability of its financial position” (8). Increasing fiscal space can be achieved through some methods, i.e. improving macroeconomic conditions, re-prioritization of health, earmarked tax, grants and efficiency. While creating “budgetary rooms” is often interpreted as generating new resources, freeing up resources through improving efficiency could be a practical and feasible way to increase fiscal space for health in countries with low resources (9).

### Health financing in Iran

Located in West Asia, Iran is an upper-middle-income country (10). In 2014, 6.9% of gross domestic product (GDP) was spent on health, while the rate of out-of-pocket payment (OOP) was 48% (11). Aiming to reach universal health coverage (UHC), since May 2014 Iran has launched Health Transformation Plan (HTP) to increase financial protection and improve equity and quality of care. Consequently, the government increased GDP for health through earmarking 1% value-added tax (VAT) and 10% of targeted subsidies for health. Despite this, providing sustainable financial resources to achieve UHC is still a challenge in Iran (12).

Iranian healthcare system is financed through different sources, mainly general government budget, insurances and out of pocket payment (OPP). Table 1 presents some health financing indicators before HTP. One of the everlasting concerns of health financing in Iran is to increase the general government expenditure on health and to reduce out of pocket payment rate. This has been addressed in several policy documents such as the Supreme Leader’s Mega Policies for the health sector and the Fifth National Development Plan. Apart from shifting from OOP to public financing, the limitation and stability of current resources are always a challenge in health sector financing. To overcome such challenges, it is crucial for the health systems to expand fiscal space for health. The aim of this article was to identify and highlight the possible options for expanding fiscal space through recognizing leakages in health system and improving efficiency in the context of healthcare system in Iran.

**Table 1:** Selected health financing indicators before the Health Transformation Plan (2014)

***Indicator***	***Percentage***
Total health expenditure as a percentage of GDP	6.89
General government expenditure on health as a percentage of Total expenditure on health	41.20
Private expenditure on health as a percentage of Total expenditure on health	58.80
General government expenditure on health as a percentage of total government expenditure	17.53
External resources for health as a percentage of total expenditure on health	0.03
Social security expenditure on health as a percentage of General government expenditure on health	31.33
Out-of-pocket expenditure as a percentage of private expenditure on health	81.30
Out-of-pocket expenditure as a percentage of total expenditure on health	47.80
Private prepaid plans as a percentage of private expenditure on health	6.67

## Materials and Methods

### Study design and data collection

We conducted a qualitative study to identify ways of expanding fiscal space for health. The study led us to recognize efficiency as one of the key potentials for improving fiscal space in healthcare system of Iran. We collected our data through face-to-face, in-depth and semi-structured interviews and developed our interview guide based on targeted literature review. Through a systematic review, we deductively identified the thematic codes regarding the potential leakage of resources. Applying principles of purposive sampling and snowball technique, 29 stakeholders from micro to macro levels of healthcare financing in Iran were selected to be interviewed. The interviews took place between Jan and May 2017, usually at the interviewees’ workplace. Interviews generally lasted between 50 and 70 min. All interviews were digitally recorded and transcribed verbatim. Table 2 demonstrates the characteristics of interviewees.

**Table 2:** The characteristics of interviewees

***Organization***	***Position***	***Number***
Parliament	Members of parliament	2
Insurance organizations	Board members and Senior officials	10
Ministry of Health and Medical Education (MOHME)	Senior officer in the budget office and in the medical tariff department	9
Universities and academic members	Professors of health financing and health policy	4
Planning and Budget Organization (PBO)	Policy makers	2
National institute for Health Research (NIHR)	Senior officials	2

### Data analysis

Thematic analysis was carried out for qualitative data analysis. We used Tandon-Cashin framework for conceptualizing fiscal space, which considers efficiency as a method of expanding fiscal space (9). We considered efficiency as the main theme of this study. By using thematic analysis we extracted 21 codes and categorized them into three subthemes. We merged some codes and ended up with 14 issues finally.

### Ethical approval

We obtained verbal consent prior to the data collection. For this study, Ethical clearance was obtained from Tehran University of Medical Sciences. (Code: IR.TUMS.SPH.REC.1395.766).

## Results

This study intended to identify the ways of wasting resources in healthcare system of Iran, that through improving them, fiscal space can be increased. We found three subthemes affecting the efficiency of healthcare system: administration, implementation and monitoring. Table 3 presents the main dimensions and their subcategories for expanding fiscal space through efficiency in Iran.

**Table 3:** Main reasons for wasting resources in healthcare system of Iran

***Main Theme***	***Subthemes***	***Issues***
Efficiency	Administration	Failure of adherence to guidelines Insufficient use of evidences in policy-making Inappropriate priority setting for allocating budget within the health system Inefficient and inappropriate use of charities Improper provider payment system Mismanagement of human resources
	Implementation	Incomplete and inefficient implementation of family physician & referral system Inappropriate strategic purchasing function Failure to implement Electronic Health Records
	Monitoring	Induced demand Overuse of medicines Overuse of para-clinic services

### Administration

There are a number of clinical and managerial guidelines mostly provided by professional groups. Some interviewees complained about lack of doctors’ appropriate compliance with the guidelines. Several reasons were mentioned for this, but the main one was inefficient administrative setting to watch out the adherence to guidelines:

*“We should link the use of guidelines to doctors’ promotion pathway. The global experience has proved that guidelines are a must in today’s healthcare systems. We can see how they get benefits of proper implementation of guidelines...”*

*(A senior health official)*

Doctors were not the only practitioners who did not comply with guidelines. Policy makers were also accused of not using sufficient evidence in deciding and executing healthcare plans, identified as a big source of wasting money:

” We begin to run a policy, then suddenly we decide to stop it. What does happen as a result of this way of decision making? Of course wasting money, wasting resources… What is the role of evidences in our country? Do we use them in policy-making process?”

*(A senior insurance official)*

Our interviewees highlighted the lack of or inappropriate priority setting as another inefficient dimension of healthcare administration in Iran, which may lead to money wasting. Most interviewees thought that setting priorities is mostly affected by political preferences and the power and influence of stakeholders, rather than being made on the basis of contextual factors, i.e. the amount of available budget, populations’ needs and the salience of problems:

“Priority setting is an important issue. For example, primary healthcare is one of the best ways of improving health outcomes with reasonable amount of money. However, historically we spend more money on secondary health care.... Though, recently the government is taking some steps to change priorities and move towards PHC.”

*(A national policy-maker)*

Donation is a good source for healthcare financing in some countries. Most healthcare managers thought that donations towards health are not efficiently used in Iran. For instance, the majority of donations go towards building hospitals in some areas, which may not be in line with the needs of that particular region. However, the MOHME has established a new deputy for social affairs, which organizes charity for health and aims to match donations with the real needs. Yet, some interviewees believed that more should be done to make the role of this office more productive within the MOHME:

“The process of using donation for health is disorganized. The office within the ministry of health should be more active than the current level to use the donations in a more targeted and specific manner.”

*(A senior health official)*

According to the interviewees’ opinion the provider payment system in Iran is one the most important sources of wasting resources that directly affect efficiency. Fee for service is the most common provider payment system in Iran. One of the interviewees believed that this system resulted in increasing supplier-induced demand:

“Fee for service is one the worst provider payment methods, because it intrinsically increase supplier-induced demand. What we need as a method of payment is diagnostic related groups (DRG)”

Many policy makers mentioned that now our main problem is the money allocated to human resources part. The amount of money we spend on this section including compensation and inefficiencies related to healthcare workers is huge and can be reduced by reorganizing the whole process:

“We spend a lot of money on human resources that can be reduced by improving human resources policies. There are a lot in this part that we can work on it. Improving indicators related to recruitment and assessment of healthcare workers, methods of payment and promotion are the most important issues that policy makers should take them into consideration.”

### Implementation

Despite the existence of good healthcare initiatives in Iran, i.e. primary healthcare (PHC), family physician and referral system, some interviewees were concerned about inappropriate or incomplete implementation of such programs. Many pointed out that efficiency gains can be achieved through increasing access (particularly in remote areas) and preventing unnecessary visits to specialists:

“People (in Iran) prefer to visit specialists. If there was a proper referral system, in which the waiting time was long (enough) to visit specialist, most probably people would have not come to visit doctors as frequently as they currently do. Big chunk of money is being wasted for this … we should move towards PHC and reorganize the referral system now more than ever. This may take some time but it is possible because we have the basics and structures.”

*(A national policy maker)*

“Our country suffers from the lack of referral system… for a problem that a midwife can handle, women go to visit a gynecologist…”

*(A senior insurance official)*

Some interviewees highlighted strategic purchasing as an effective method to curb the costs of healthcare systems. Strategic purchasing is not being practiced well in Iran and requested it to become a necessity for Iran’s healthcare financing system:

“We need to look at other countries’ experiences in this regard… Thailand is a good example, whose success is rooted in strategic purchasing” (A national policy maker) “Strategic purchasing should be implemented (in Iran) more seriously. It gives us options to improve the efficiency of our system”

*(A national policy maker)*

In addition, some interviewees emphasized the implementation of electronic health records (EHRs) to enhance healthcare efficiency in Iran. Despite implementing EHRs for the majority of population, most interviewees were concerned about meaningful, effective and comprehensive use of EHRs to improve quality and safety of healthcare in the country.

“*We don’ have clear and detailed information of our covered population. We do not know for what and how often people use the services. We need to promote EHR system”*

*(A national policy maker)*

For instance, some interviewees had a view that EHRs may reduce cost of healthcare in many ways including preventing overuse or misuse of service as well as increasing patient safety:

“People are entitled to use services that they do not really need or might cheat on the system just because there is no overarching system of information. If every person would have a personal electronic card including their detailed health information, we could have prevented most wastages”

*(A senior health financing officer)*

Most policy makers also criticized the way of managing the medicines’ market in Iran that led to considerable wastage of resources and addressing this may bring more efficiency to healthcare system:

“Our medicine market is chaos and there are a lot of wasting. It is accepted in the world that this part of all healthcare systems in the world suffers from a lot of wasting. We need to focus more on this area. (A senior health financing officer) “The previous Government suddenly added 253 items to the benefit package without any evaluation”

*(A national policy maker)*

### Monitoring

Monitoring is a confirmed method of decreasing costs. A number of our interviewees in managerial roles mentioned that Iran’s healthcare system has a long way to go in achieving an effective monitoring system. Three interrelated dimensions including induced demand, overuse of medicines and overuse of para-clinic services were emerged in this regard.

“We suffer from a good monitoring system and due to this we lose resources that can be used in a more efficient way. The golden key to improve our efficiency is to have more control over using resources”

*(A senior finance officer)*

One dimension was lack of control on both provider and purchaser’s behavior to prescribe, use and purchase healthcare services in Iran. For instance, interviewees accused doctors of prescribing diagnostic tests and medicines easily without any limit or without any proper indication showing patients’ need. Worse still, patients sometimes demand more prescriptions to get medicines or do lab tests despite the fact that they do not need them, just because their insurance covers it. Unfortunately, there is no conventional practice for effective monitoring and control of both providers and users of services towards rational prescription and use of healthcare services. Some interviewees recommended a functioning monitoring system to prevent unnecessary use of medicines and other healthcare services in Iran:

“Our patients ask doctors to prescribe MRI, CT scans, various medicines, e.g. antibiotics for them… This is rather a cultural problem. We need to educate our people and inform them about the cost and safety concerns that their (irrational) behavior imposes to the healthcare system. For example, irrational use of antibiotics has led to antimicrobial resistance, which in turn will waste much more of our money.”

*(A senior health insurance official)*

## Discussion

This study aimed to shed light on reasons that may result in wasting resources within the healthcare system in Iran, identifying and preventing which can improve efficiency and expand fiscal space for health. We found a few potential spaces not considered as of high priority in health financing context, considering which may strongly expand fiscal space for healthcare system in Iran. We categorized them under efficiency title and explained their relation to resources wasting.

Resource constraints have been a longstanding challenge in nearly all countries, especially in the low and middle-income settings (13). Inefficient use of current resources and wastage are common problems in many healthcare systems. Inefficiency is responsible for 20% to 40% of waste in all health spending (14). Nevertheless, getting the most out of current resources is a sensible way to tackle such a challenge (2). Countries need to cut costs in some points and consequently free up fiscal space through utilization of many capacities that are currently being ignored. Not only efficiency is the only way to fill the financial gaps of healthcare system, but actually, it can be one of the most essential ways of enhancing fiscal space (8, 15). Efficiency can improve outcomes and reduce costs (16). Evidence emphasizes that achieving a reasonable level of efficiency is at the heart of reviving healthcare financing in any setting (17, 18).

Healthcare systems’ inefficiency is rooted in different causes. Cutting the costs of healthcare might not be necessarily equal to moving towards efficiency (2). Rather, an organized system that applies appropriate methods, may reduce waste and increase the best use of current resources, hence improving efficiency.

Clinical guidelines are developed to provide pathways aiming to help physicians for evidence-based diagnosis and treatment of diseases (19). Our study reconfirmed the lack of adherence to clinical guidelines in Iran. The rate of adherence is different in various contexts, but overall rate in Iran is lower than the global trend (20). Adherence to clinical guidelines can reduce costs by improving and preventing overuse of diagnosis tests and improving decision making process. Likewise, evidence-based policy in a way that evidence is used irrespective of political preferences to make the right choices, is crucial to gain efficiency (21).

There are few studies that confirm the inefficient use of donation for healthcare system in Iran (22). The literature is not rich in this area and therefore more research is required to show the gap. In terms of donations, most donors follow the purposes that are not necessarily aligned with healthcare needs.

There are studies that show increasing fiscal space for health is possible through changing the provider payment system mainly from fee for service to other methods according to the possibility and feasibility of applying a method and countries’ policies (17, 18).

The other issue which is of high priority in improving efficiency is improving human resources policies. Some countries like Nepal and Uganda have set policies in this regard to improve their efficiency (18, 23).

Appropriate and complete implementation of PHC in healthcare initiatives can contribute to healthcare efficiency. A well-established and strong PHC can improve healthcare systems’ performance in various ways, particularly with handling the simple issue in first level and preventing unnecessary visits to specialist. There are a number of advantages to using PHC and the most important one is facilitating the implementation of family physician and consequently referral system (24, 25).

Strategic purchasing is one of the main health financing instruments to improve access to healthcare and consequently health outcomes (26). Changing the approaches from passive to active purchasing (strategic) can improve health systems’ performance, as also most interviewees acknowledged (27, 28).

EHRs can prevent excessive costs in healthcare systems by helping health policy-makers to have sufficient information for policy making (29). EHRs can provide a platform to hold accurate and up-to-date information about covered population and their use, overuse or misuse of healthcare services, hence contributing to avoid moral hazard as well as preventing medical errors. Appropriate use of EHRs can save medical staffs’ time and boost efficiency in financial terms (30–33).

Finally, appropriate and customized monitoring system is a key to avoid significant financial burden due to overuse of healthcare services and medicines, particularly in Iran, where overuse of services tends to be higher than average (34, 35). Inappropriate monitoring may also lead to induced demand by service providers who seek to increase their income (35). The important point is that preventing or controlling this issue is relatively hard. The government needs a very high standard monitoring system that decrease health expenditures.

### Policy Implications

To tackle several existing inefficiencies deriving from various sources, health financing system in Iran needs to improve efficiency. The legislative body can adopt appropriate policies to take more out of the current resources. The executive body needs to enhance its supervision over the policy implementation process. All in all, since healthcare spending has reached a historical high in Iran, there is still room to improve efficiency for expanding fiscal space for health through the existing resources.

## Conclusion

Limited resources is a challenge that many countries are to some extent facing. In response to this challenge, it is not necessary to generate new resources. Rather, freeing up fiscal space through efficiency looks like to be a feasible option. To improve efficiency, every healthcare system should identify the leakages and respond to the wastage through appropriate planning. Getting the most out of current resources is possible through implementing tailored and evidence-based policies or programs, proper administration and finally a well-established monitoring system. While the health system in Iran is moving towards UHC along the pathway of SDGs, it is necessary, now more than ever, to expand fiscal space and strengthen the healthcare system to respond to ever-increasing demand.

## Ethical considerations

Ethical issues (Including plagiarism, informed consent, misconduct, data fabrication and/or falsification, double publication and/or submission, redundancy, etc.) have been completely observed by the authors.
